# EphA2-Receptor Targeted PEGylated Nanoliposomes for the Treatment of BRAF^V600E^ Mutated Parent- and Vemurafenib-Resistant Melanoma

**DOI:** 10.3390/pharmaceutics11100504

**Published:** 2019-10-01

**Authors:** Yige Fu, Drishti Rathod, Ehab M. Abo-Ali, Vikas V. Dukhande, Ketan Patel

**Affiliations:** Pharmaceutical Sciences, St. John’s University, Queens, NY 11439, USA

**Keywords:** EphA2 receptor, PEGylated nanoliposomes, melanoma cancer, trametinib

## Abstract

The clinical outcomes of malignant melanoma have improved with the introduction of mitogen-activated protein kinase kinase (MEK) inhibitors. However, off-target toxicities of the MEK inhibitor trametinib (TMB) often result in dose interruption and discontinuation of therapy. The purpose of this study was to anchor a physically stable EphrinA1-mimicking peptide known as YSA (YSAYPDSVPMMS) on TMB-loaded PEGylated nanoliposomes (YTPLs), and evaluate them in BRAF^V600E^-mutated parent cells (lines A375 and SK-MEL-28) and vemurafenib-resistant cells lines (A375R and SK-MEL-28R) in melanoma. TMB-loaded PEGylated liposomes (TPL) functionalized with nickel-chelated phospholipids were prepared using a modified hydration method. The hydrodynamic diameter and zeta potential values of optimized YTPL were 91.20 ± 12.16 nm and –0.92 ± 3.27 mV, respectively. The drug release study showed TPL did not leak or burst release in 24 h. The hemolysis observed was negligible at therapeutic concentrations of TMB. A differential scanning calorimetry (DSC) study confirmed that TMB was retained in a solubilized state within lipid bilayers. YTPL showed higher intracellular uptake in parental cell lines compared to vemurafenib-resistant cell lines. Western blot analysis and a cytotoxicity study with the EphA2 inhibitor confirmed a reduction in EphA2 expression in resistant cell lines. Thus, EphA2 receptor-targeted nanoliposomes can be useful for metastatic melanoma-specific delivery of TMB.

## 1. Introduction

Melanoma is a malignant tumor formed from melanocytes (pigment-producing cells), and is the sixth most common type of cancer in the United States [1]. Approximately 80% of the melanomas are diagnosed at localized stages, and one-third of those early-stage patients develop metastatic melanoma. The survival rate is less than 10% for the patients with metastatic melanoma [2]. The introduction of BRAF-inhibitors like vemurafenib and dabrafenib and MEK-inhibitors (MEKi) like trametinib (TMB) has resulted in significant improvement in clinical outcomes; such therapeutic regimens have remarkably changed the survival statistics of malignant melanomas in recent years [3,4,5]. However, off-target toxicities often lead to dose interruptions, restrict dose escalation, and warrant reductions in the dose of BRAFi and MEKi, especially trametinib. Dose-dependent side effects associated with TMB include: an absolute decrease of >10% in left ventricular ejection fraction (LVEF), ocular and interstitial lung disease, rash, hypertension, fatigue, peripheral edema, diarrhea, and acneiform dermatitis [6]. Management of decreased LVEF requires withholding TMB doses for up to four months, or in severe cases, permanent discontinuation of TMB. Central serous retinopathy and hypertension also require dose modification. It is quite apparent that reduction in the dose will negatively affect the clinical outcome of the chemotherapy [7,8]. Therefore, drug delivery to the desired site is preferred for metastatic melanoma to reduce adverse drug effects. 

Delivery of anticancer drugs via active and passive targeting has been studied widely using nanoparticulate systems [9]. An enhanced permeability and retention (EPR) effect in solid tumors is becoming a gold standard in antitumor drug delivery, and helps in passive targeting due to the leaky vasculature and abnormal lymphatic drainage system. Various researchers have demonstrated the benefits of EPR effects in enhancing tumor-specific distribution/preferential uptake and improving the efficacy of nanoparticles and theranostic [10,11,12]. Due to the EPR effect, nanoliposomes reach the tumor site by passively targeting the delivery of drugs. In addition, active targeting of cancer cells can be achieved by attaching/anchoring the ligand (e.g., folic acid, Arginylglycylaspartic acid (RGD), glucosamine, etc) that would be directed to overexpressed receptors on cancer cells [13,14,15]. Among the distinct nanoparticulate systems that have been investigated to date, liposomal carriers have proven to be advantageous over other types of nanocarriers. Their unique structure and the safety and biocompatibility of the phospholipid excipient make them superior to non-ionic surfactants and polymers for intravenous delivery. Further, surface PEGylation of nanoparticles is a preferred method in order to bypass the reticuloendothelial system and proteolytic enzymatic degradation [16,17].

Recent studies demonstrated the pivotal role of overexpressed EphA2 receptors in aggressiveness, metastatic potential, and vasculogenic mimicry in malignant melanoma [18,19]. EphA2 is also considered a growth receptor for malignant melanoma [20]. Melanoma tumor samples from patients taken prior to or after treatment with BRAFi and MEKi showed very high expression of EphA2 in both BRAF^V600E^ and BRAF^WT^ melanomas [20]. Compared to the EphA2 expression in malignant melanoma, normal tissues have low EphA2 expression, and therefore using YSA peptide-anchored nanocarriers for drug delivery may reduce off target side effects by reducing exposure of cytotoxic drugs to normal cells [21]. Moreover, upregulation of the EphA2 receptor was observed in BRAFi-resistant cell lines (such as A375 and SK-MEL-28) [22]. Thus, the cell surface EphA2 receptor could be a potential target not only for treatment but also for delivering a high payload of anti-melanoma drugs. Wu et al. demonstrated that YSA-conjugated paclitaxel was more effective in murine melanoma compared to paclitaxel alone [23]. Moreover, YSA-anchored nanocarriers can carry a much higher load of a drug to the tumor compared to individually conjugated molecules [24]. To our knowledge, there have been no studies demonstrating EphA2 receptor-targeted nanoparticles with anticancer agents for metastatic melanoma. We hypothesized that anchoring of the EphrinA1-mimicking peptide YSA (YSAYPDSVPMMS) on the surface of TMB-loaded nanoliposomes (YTPLs) would selectively target the EphA2 receptor on metastatic melanoma cells and deliver a high payload of TMB in tumor tissues [25,26,27]. Surface PEGylation makes stealth nanoliposomes from the reticuloendothelial system (mononuclear phagocyte system) of the lung, liver, spleen, and kidney [28]. Moreover, a long circulation of nanoliposomes facilitates accumulation in the tumor matrix while active targeting promotes internalization [29]. This drug delivery strategy can significantly limit the amount of free TMB in blood, and as a result, minimizes the adverse effects. We hypothesized that long circulation of PEGylated nanoliposomes facilitates their accumulation in the melanoma tumor matrix, and specific delivery of TMB would minimize off-target side effects by limiting the availability of free TMB in circulation. The specific objective of this paper was to develop and characterize YTPL using a modified hydration method in BRAF^V600E^-mutated parent and resistant melanoma cell lines.

## 2. Materials and Methods

### 2.1. Materials

Trametinib was purchased from LC Laboratories (Woburn, MA, USA); 1,2-Dioleoyl-sn-glycero-3 phosphocholine (DOPC) was purchased from Cordenpharma (Liestal, Switzerland); PE 18:0/18:0-PEG2000 was obtained from Lipoid (Ludwigshafen, Germany); cholesterol, chloroform, and a Sephadex G50 column were purchased from Sigma-Aldrich (St. Louis, MO, USA); 6-histidine tagged PEGylated (PEG) YSA (6His-PEG-YSA) was obtained from GenScript Corporation (Piscataway, NJ, USA); DOGS-NTA-Ni was obtained from Avanti (Alabaster, AL, USA); Fetal Bovine Serum (FBS) was procured from Atlanta Biologics (Oakwood, GA, USA); Dulbecco’s modified Eagle’s medium (DMEM) and the BCA protein estimation kit were purchased from ThermoFisher Scientific Inc. (Waltham, MA, USA); Penicillin–Streptomycin–Amphotericin B (PSA) was purchased from MP Biomedicals, LLC (Solon, OH, USA); and ALW-II-41-27 was obtained from Cayman Chemical (Ann Arbor, MI, USA). Other chemicals and solvents were of analytical grade. Melanoma cell lines (A375 and SK-MEL-28) were obtained from American Type Culture Collection (Manassas, VA, USA).

### 2.2. Analytical Method

Chromatographic separation of TMB was achieved using Waters e2695 separation module, a 2998 PDA detector instrument equipped with a Hypersil ODS C18 column (250mm × 4.6 mm, 5 μm). Acetonitrile:phosphate buffer pH 3.5 (70:30) was used as the mobile phase with a flow rate of 1 mL/min and an injection volume of 10 µL. The column temperature was kept at 25 °C and the output signal was detected using Empower 3 software. The retention time of TMB was found to be 4.7 min, detected at 248 nm. HPLC standard curve of TMB is provided as supplementary file – Figure S1.

### 2.3. Cell Culture

Vemurafenib-resistant cell lines (A375R and SK-MEL-28R) were generated by adding 0.2 µM of vemurafenib to the media of A375 and SK-MEL-28 for 2 months. The vemurafenib resistance was confirmed prior to studies. All cell lines were maintained in DMEM, supplemented with 10% FBS and 1% PSA, and incubated at 37 °C with 5% CO_2_.

### 2.4. Preparation of Liposomes

Initially, liposomes were prepared by different methods. Briefly, TMB:DOGS-NTA-Ni:DOPC:cholesterol:DSPE-PEG 2000 in a 1:0.75:60:16.3:2.1 molar ratio were dissolved in chloroform. DOPC, cholesterol, DSPE-PEG, and TMB were dissolved in chloroform. For thin film hydration, the solution was taken in a round bottom flask under vacuum to form a film, followed by hydration with water at 55 °C and ultrasonication (30% amplitude) for 2 min. For the modified hydration method, the same chloroform solution was added drop-wise to parenteral-grade mannitol (200 μm) with constant stirring at 45 °C and left overnight for evaporation of chloroform. Dispersion of this resultant powder was prepared in water at 55 °C and was sonicated (30% amplitude) for 2 min. For the investigation of the effect of mannitol on stability, mannitol was separated from liposomes using a Sephadex G50 column. For preparation of YTPL, first DOGS-NTA-Ni-loaded liposomes were prepared using the same composition and method described above. Liposomes were incubated with different concentrations of YSA for 30 min to obtain liposomes with varied molar ratios of DOGS-NTA-Ni:YSA.

### 2.5. Characterization of Liposomes

The average size, size distribution by intensity, zeta potential, and polydispersity index (PDI) were measured using a dynamic light scattering (DLS) particle size analyzer (Malvern Zetasizer Nano ZS, Royston, UK). Samples were analyzed using disposable cuvettes at 25 °C with a scattering angle of 173°. The effect of YSA concentration on particle size and zeta potential was evaluated. All the experiments were carried out in triplicates. Entrapment efficiency was estimated using ultrafiltration by Amicon ultra centrifugal filters (50K). The concentration of TMB was analyzed by HPLC. The encapsulation efficiency of TMB was expressed as the percent of drug encapsulated and calculated using the following formula:

Percent encapsulated = ((Total TMB) – (Free TMB)) / (Total TMB) × 100%.(1)

### 2.6. Stability Study

TPL at different drug loading values (1%, 2.5%, and 4%) was prepared for the stability study. Total drug content was measured at time zero. Samples were withdrawn at different time points to analyze the amount of precipitated drug by HPLC.

### 2.7. Freeze Drying of Liposome

TPL with 1% drug loading was used to investigate the effect of concentration of cryoprotectant (2.5%, 5%, 7.5%, 10% trehalose) on freeze drying. TPL was prepared using the method described above (unfiltered TPL). Further, unfiltered TPL was passed through a Sephadex G50 gel column to separate mannitol. Mannitol-free TPL was refered to as filtered TPL. Briefly, 1-mL aliquots of the liposomal dispersions were filled into colorless glass vials and then stored at −80 °C overnight, followed by lyophilization (Labconco FreeZone 2.5, 53 °C at 12 Pa) overnight in order to achieve a preservable white powder. The same protocol was used for freeze drying of YTPL. Lyophilized liposomes were reconstituted with water. Particle size, zeta potential, and YSA binding efficiency were analyzed before and after freeze drying of YTPL. For YSA binding efficiency, 400 µL of YTPL were filled into Amicon Ultra centrifugal filters (30 kDa) (Millipore, Ireland). Free YSA was separated by centrifuging the samples at 10,000 rpm for 10 min. Concentration of free YSA (before and after freeze drying) was analyzed using a BCA protein estimation kit. (method details given in supplementary file 1).

%YSA binding = (amount of YSA attached to liposomes/total YSA added) × 100 (2)

### 2.8. Differential Scanning Calorimetry (DSC) Thermograms of TMB and TPL

Analysis of TMB and TPL was carried out to evaluate the physical state of TMB in liposomes using a Q200 modulated DSC instrument (TA Instruments, New Castle, DE, USA). The liposomal formulation was dried, and the semi-solid paste was weighed in an aluminum pan and hermetically sealed. The samples were equilibrated at 25 °C for 5 min and were heated at the rate of 10 °C/min from 40 °C to 350 °C. A hermetically sealed empty aluminum pan was used as a reference. TA Instruments Universal Analysis 2000 software (TA Instruments) was used to analyze the data.

### 2.9. In Vitro Release Study

Drug release from TPL was carried out using the dialysis bag method. Before use, dialysis bags (Spectra/Por^®^ 7) were soaked before use in Milli-Q water at room temperature overnight to remove the preservative, followed by rinsing thoroughly in Milli-Q water. Drug release of TMB from TPL was carried out in a dialysis sac with 200 mL of phosphate buffer saline (pH 7.4) containing 0.5% Kolliphor EL at 37 °C with constant stirring. The samples were withdrawn from the release medium at different time intervals. The amount of TMB in the release media was evaluated by HPLC.

### 2.10. In Vitro Hemolysis Study

Rat red blood cells (RBCs) were separated from plasma by centrifugation at 2000 rpm for 5 min. The cell pellet was re-dispersed into an appropriate volume of PBS to achieve the same hematocrit. Then, 1 mg/mL TMB of TPL was added to the RBC dispersion to achieve 100, 50, 10, and 2 μg/mL TMB concentrations. After 30 min of incubation at 37 °C, samples were centrifuged at 2000 rpm for 10 min. Supernatants were diluted with PBS and analyzed. A UV spectrophotometer was used to evaluate the hemoglobin release at 550 nm. PBS was used as the negative control, and sodium lauryl sulfate solution was used as the positive control (100% hemoglobin release). Percentage hemolysis was calculated by following formula:

% hemolysis = (absorbance of test sample – absorbance of negative control)/ (absorbance of positive control – absorbance of negative control) × 100 (3)

### 2.11. Plasma-to-Blood Ratio

TPL was added to blood to prepare TMB at a concentration of 50 μg/mL (*n* = 6). Samples were incubated at 37 °C for 30 min and then samples were centrifuged. Plasma was separated from centrifuged samples. Sodium lauryl sulfate was added to half the samples for complete hemolysis. The TMB concentration in haemolyzed blood and plasma was analyzed by HPLC. The plasma-to-blood ratio was calculated by the equation given below:

CB/CP = Concentration of TMB in whole blood/Concentration of TMB in plasma (4)

### 2.12. Cellular Uptake of Liposomes

Cells were plated in a 96-well plate at a density of 10,000 cells/well and incubated at 37 °C and 5% CO_2_ for 48 h before treatment. Coumarin-6 loaded PEGylated liposomes (C6PL) and YSA-anchored coumarin-6-loaded PEGylated liposomes (YC6PL) were incubated with cells for 1 h. Afterwards, cells were washed with HBSS and fixed with 3.7% formalin. Uptake of TPL and YTPL in different cell lines was observed for same exposure time using the EVOS FL Auto Cell Imaging System with 40× magnification.

### 2.13. In Vitro Cytotoxicity Test

Cytotoxicity of TMB, TPL, and YTPL was evaluated in A375, SK-MEL-28, A375R, and SK-MEL-28R cell lines using 3-(4,5-dimethylthiazol-2-yl)-2,5-diphenyl tetrazolium bromide (MTT) assay. ALW-II-41-27 (an EphA2 receptor ATP-competitive inhibitor), vemurafenib, and vemurafenib with 0.1 µM ALW-II-41-27 were tested in both SK-MEL-28 and SK-MEL-28R. Cells were seeded in 96-well plates at a density of 5000 cells/well and allowed to grow for 24 h before treatments. TMB and TPL were diluted in cell culture medium at different concentrations. After 48 h treatment, cell viability was determined by the MTT assay. Briefly, MTT dye was dissolved at a final concentration of 5 mg/mL in PBS. Cells were incubated with 20 μL of 5 mg/mL MTT solution in each well for 3 h at 37 °C, 5% CO_2_. Then the medium were removed from wells and MTT-formazan crystals were dissolved by the addition of 100 μL of dimethyl sulfoxide (DMSO) to each well. The quantity of MTT-formazan was determined by 570 nm absorbance as the wavelength reference.

### 2.14. Western Blot Assay

Whole cell protein lysates were obtained from A375, SK-MEL-28, A375R, and SK-MEL-28R cell lines. Briefly, cells were scraped in modified RIPA buffer (50 mM Tris, 150 mM NaCl, 1% *v/v* NP-40, 0.5% *w/v* deoxycholate, 0.1% *w/v* SDS, 10% *v/v* glycerol, 10 mM NaF, 0.4 mM EDTA, pH 8.0) with protease inhibitors. The lysates were cleared by centrifugation at 10,000 g for 10 min and then reduced with Laemmli buffer containing β-mercaptoethanol, separated on 4–15% MiniProtean TGX gels (Bio-Rad, Deesid, UK), transferred to a PVDF membrane, and probed with primary antibodies from Cell Signaling Technology for EphA2 (6997) and *β*-actin (8457) for chemiluminescence detection using the Azure Biosystems c500 imager (Dublin, CA, USA).

### 2.15. Statistical Analysis

Each experiment has been performed in triplicate and carried out at least three times. All statistical analyses were performed using Student’s *t*-test or one-way ANOVA using GraphPad Prism7 (GraphPad Software, La Jolla, CA, USA) and Microsoft Excel. Differences between groups were considered to be significant at a *p-*value of <0.05. 

## 3. Results

### 3.1. Preparation of TMB-Loaded PEGylated Liposomes

The results of TPL prepared using different methods are given in Table 1. Due to the poor solubility of TMB in ethanol and ether, the ethanol injection method and ether injection method were not used for the preparation of TMB liposomes. Thin film hydration method is a commonly used method for the preparation of liposomes. However, around 50% of the drug precipitated from TPL in 2 h. Moreover, entrapment efficiency was only 51.6%. Because of the poor entrapment and physical stability of TPL prepared using a thin film hydration method, we adopted a modified hydration method, which showed 96.2% entrapment of TMB and better physical stability (absence of TMB precipitation within 24 h) compared to the thin film hydration method. Thus, a modified hydration method was used for further development of YSA-anchored trametinib-loaded PEGylated nanoliposomes (YTPL).

### 3.2. Particle Size and Zeta Potential

**Figure 1:**
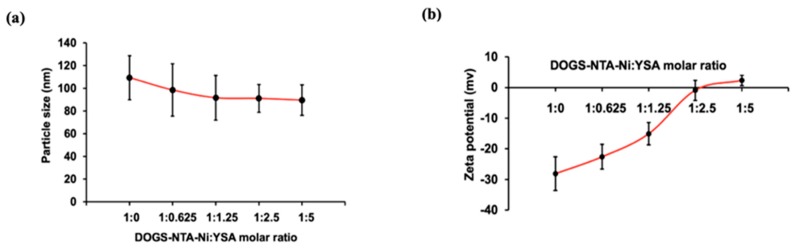
Effect of YSA (YSAYPDSVPMMS) concentration on (**a**) particle size and (**b**) zeta potential. Data are given as mean ± SD (*n* = 3). No significant change in particle size while zeta potential increased as YSA concentration increased.

The hydrodynamic diameter of TPL prepared using a modified hydration method was found to be 109.45 ± 9.40 nm with a zeta potential of −35.55 ± 9.60 mV. The zeta potential of DOGS-NTA-Ni-loaded liposomes was lower than TPL. For YTPL, particle size was found to be similar at all the DOGS-NTA-Ni: YSA ratios (Figure 1a). A slight decline in particle size from 109.45 nm to 89.75 nm was observed after incubation with YSA. An increase in the zeta potential of liposomes was observed in a concentration-dependent manner due to the surface complexation of YSA (Figure 1b). The zeta potential significantly increased from −28.10 mV to −0.92 mV upon addition of 1:1.25 M DOGS-NTA-Ni: YSA. Thereafter, there was only a slight increase in zeta potential at higher ratios (e.g., 1:2.5 and 1:5).

### 3.3. Stability of Liposomes

**Figure 2:**
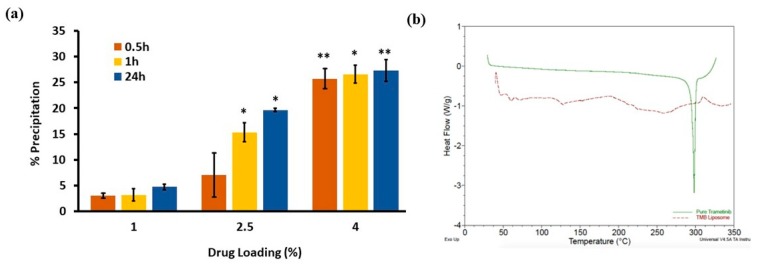
Stability study and solid-state characterization of TPL. (**a**) % precipitation of TPL with varying drug loading values. Precipitation significantly increased with higher drug loading compared to 1% drug loading. *p < 0.05 and **p < 0.01. Data are given as mean ± SD (*n* = 3). (**b**) Differential scanning calorimetry (DSC) thermograms of TMB and TPL. Green peak showed a crystalline form of TMB; the absent peak of the red line showed TMB was in soluble state in liposome.

The precipitation of a hydrophobic drug from liposomes is another issue with respect to long term stability. In order to evaluate the physical stability, TPL with different drug loading values (1%, 2.5% and 4%) at a 0.5 mg/mL TMB concentration were prepared. As expected, we observed that lower the drug loading, the lower the percentage of TMB precipitation (Figure 2a). Moreover, the precipitation increased with time. For TPL with 4% drug loading, more than 25% of the drug precipitated within half an hour, while at 2.5% drug loading of TPL, the precipitation was slower compared to 4%. However, more than 14% of the drug precipitated in 1 h. An increase in percentage precipitation with time suggested that TPL in liquid form may not be stable for long periods. Thus, considering the poor physical stability of TPL in liquid form, freeze drying was carried out. TPL with 1% loading was considered for freeze drying. Moreover, we investigated the effect of storage conditions (room temperature and 4 °C) on liposome stability. For 4% and 2.5% drug loading, precipitation was significantly affected by storage conditions. For 1% drug loading, precipitation was less than 5% at room temperature and 4 °C in 24 h (Data not shown). 

### 3.4. Freeze Drying of Liposomes

**Figure 3:**
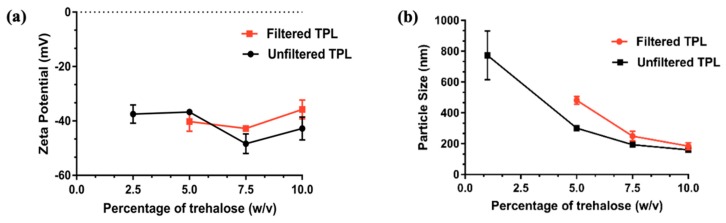
Effect of trehalose concentration of TPL on (**a**) Zeta potential (**b**) Particle size after freeze drying. Trehalose concentration has a major effect on particle size and a minor effect on zeta potential. Data are given as mean ± SD (*n* = 3).

Freeze drying was carried out to covernt TPL into solid powder for reconstitution. Since TPL was prepared using a modified hydration method that contains mannitol, we evaluated the effect of mannitol on TPL stability. Unfiltered TPL are liposomes without the separation of mannitol. Filtered TPL was prepared by separating mannitol using a G50-Sephadex column while the unfiltered TPL was the liposme which had not undergone filtering of the mannitol. Particle size and zeta potential of unfiltered and filtered TPL before and after freeze drying with various concentrations of trehalose are depicted in Figure 3 a and b. We observed that concentration of trehalose plays a signifincat role in the particle size of reconstituted TPL. Batches with 5% and 7.5% of trehalose exhibited a paste-like appearance with poor flow properties, and took a longer time for reconstitution. TPL with 10% trehalose gave free flow powder and could be reconstituted within 5 min with the original particle size and zeta potential. For TPL with 2.5% trehalose, reconstitution could not be achieved. Therefore, 10% trehalose was used for freeze drying of optimized YTPL. Freeze-dried liposome contains 2.83 µg TMB/mg of powder. No significant change of zeta potential was observed, as shown in Table 2. Particle size was increased but was still within 200 nm. Moreover, the encapsulation efficiency of YTPL remains the same (>96%) as before freeze drying. No significant different of YSA binding percentage was observed before and after freeze drying, which is complementary to the result of zeta potential.

### 3.5. DSC Thermograms of TMB and TPL

DSC endotherms of TMB and TPL were obtained as shown in Figure 2b. TMB showed a sharp endothermic peak of pure TMB at 300 °C, which suggests that the pure TMB was in a crystalline form. As expected, liposomes did not show any melting endothermic peak. Therefore, the absence of a sharp endothermic peak at 300 °C in liposomes confirmed that TMB was not in a crystalline or precipitated state but was in a solubilized state within lipid bilayers.

### 3.6. Drug Release Study

**Figure 4:**
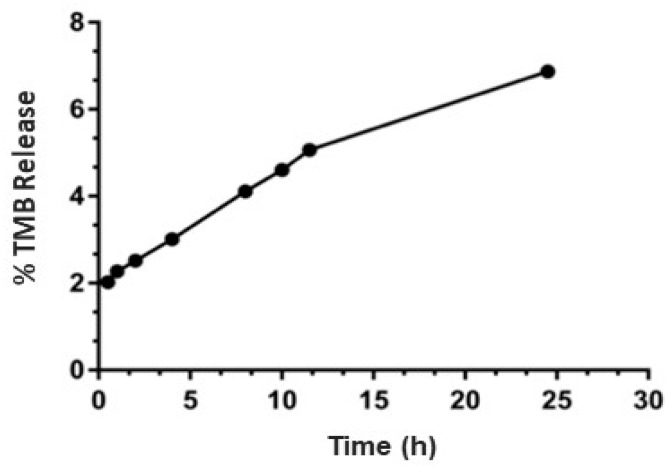
In vitro release study of TPL. No hemolysis was observed. Limited amount of release in pH 7.4 at sink condition. Data are expressed as mean and standard deviation.

TPL showed less than an 8% drug release in 24 h at sink conditions (Figure 4), which indicated that TPL did not leak or have burst release. We anticipate that TPL will follow a similar release behavior in vivo. Sink condition was maintained by adding a non-ionic surfactant kolliphor EL in the release medium.

### 3.7. In Vitro Hemolysis Study and Plasma to Blood Ratio

**Figure 5:**
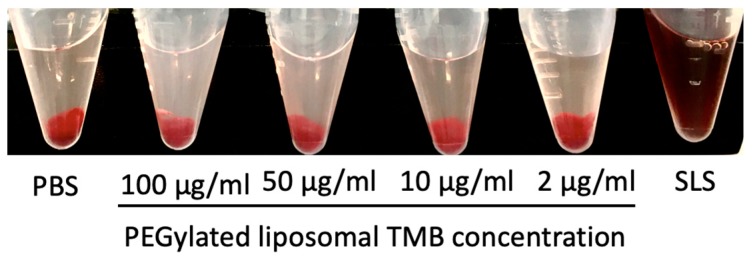
In vitro hemolysis study of TPL at various TMB concentrations.

Negligible hemolysis was observed even at 100 μg/mL of TMB used (Figure 5, Table 3). Further, very quick and complete redispersion of red blood cells (RBCs) implied that the surface characteristics of RBCs were not altered by TPL. The calculated value of the plasma-to-blood ratio for TPL was 1.01.

### 3.8. Uptake Study

**Figure 6:**
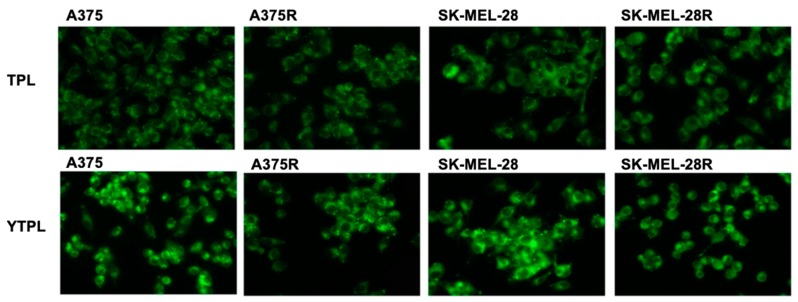
Uptake study of TPL and YTPL of BRAF^V600E^-mutated parent and vemurafenib-resistant melanoma cell lines. High intensity of fluorescence was observed with targeted liposome treatment and resistant cell lines showed less uptake of liposomes. Images were captured at 20× magnification.

Coumarin-6 was selected as the fluorescent dye for labelling the liposomes. As shown in Figure 6, the intensity of green fluorescence was significantly higher in YTPL-treated cells compared to TPL-treated cells due to the targeting peptide. Moreover, A375 and SK-MEL-28 cell lines showed higher intracellular fluorescence intensity than A375R and SK-MEL-28R for YTPL due to the higher expression of EphA2 receptors.

### 3.9. In Vitro Cytotoxicity Test

In vitro cytotoxicity of TMB, TPL, and YTPL was evaluated in patent cell lines only. IC_50_ values of each of the formulations is given in Table 4. The IC_50_ values for TMB, TPL, and YTPL were similar at around 0.7 nM. Cell viability graphs of TMB, TPL, and YTPL in melanoma cell lines are given in supplementary file – Figure S2.

**Figure 7:**
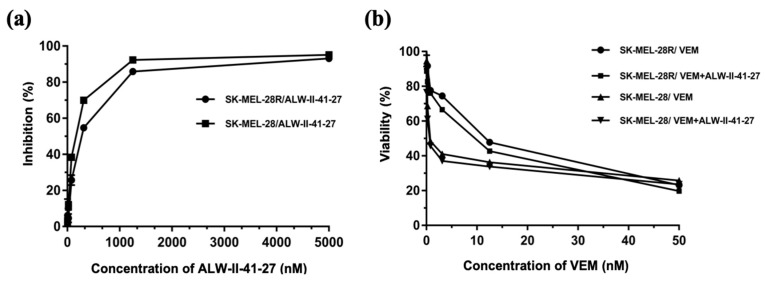
Cytotoxicity assay in parent and vemurafenib-resistant melanoma cell lines (**a**) % Cell growth inhibition of SK-MEL-28 and SK-MEL-28R after ALW-II-41-27 treatment (**b**) % Cell viability of SK-MEL-28 and SK-MEL-28R after vemurafenib treatment with and without ALW-II-41-27. Vemurafenib was incubated with 0.1 µM ALW-II-41-27. Results showed no difference in viability compared to a single vemurafenib treatment alone.

To further investigate whether the EphA2 receptor was correlated with vemurafenib-resistance, ALW-II-41-27, an EphA2 ATP-competitive inhibitor, inhibited the growth of SK-MEL-28 and SK-MEL-28R in a concentration-dependent manner (Figure 7). The IC_50_ values of ALW-II-41-27 were 122.4 nM and 177.0 nM for SK-MEL-28 and SK-MEL-28R, respectively. Co-treatment with ALW-II-41-27 did not alter the cytotoxicity of vemurafenib in parent or vemurafenib-resistant SK-MEL-28 cells. Vemurafenib diluted in 0.1 µM ALW-II-41-27 showed similar viability compared to single vemurafenib treatment, which indicates that the EphA2 inhibitor did not change the resistance sensitivity. Thus, EphA2 receptor inhibition is not co-related to cytotoxicity of vemurafenib or vemurafenib resistance in melanoma.

### 3.10. Western Analysis

**Figure 8:**
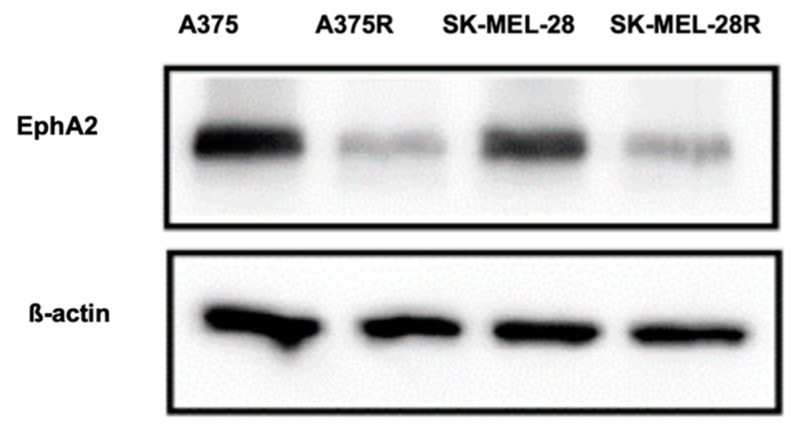
Results of expression of EphA2 receptor were determined by Western blot assay. Higher EphA2 receptor expression was observed in BRAF^V600E^-mutated cell lines than in the vemurafenib-resistant cell line (*n* = 3).

The protein expression of EphA2 was significantly lower in the whole cell lysates from A375R and Sk-MEL-28R melanoma cell lines compared to A375 and Sk-MEL-28 (Figure 8). The results further confirm that the expression of EphA2 receptor is higher in BRAF^V600E^-mutated melanoma parent cell lines compared to the resistant cell lines.

## 4. Discussion

Toxicities associated with the use of oral MEKi (e.g., trametinib and cobimetinib) severely restrict the targeted therapy of malignant melanoma. The aim of this paper was to investigate whether an EphA2 receptor-directed liposomes could be helpful in targeting parent and BRAFi-resistant melanoma. For the first time, we developed EphA2 receptor-targeted TMB-loaded PEGylated nanoliposomes to minimize the off-targeted side effects of TMB. We further characterized the liposomes for cellular internalization in parent and vemurafenib-resistant melanoma cells.

Nanotechnology has had a profound effect on many areas of healthcare and scientific research, the use of tumor-targeted nanocarriers allow for the delivery of low water-soluble cancer medications to selectively bind cancer cells and minimize off-target side effects [30,31]. The third-generation liposomes feature active-targeting and physicochemical targeting functions besides possessing a passive targeting via the EPR effect. Many papers have illustrated the important role of tumor antigen targeted-liposomes in diagnostic or treatment in cancer [32,33,34]. The active-targeting liposome includes surface attachment of small molecular ligand, peptide, or monoclonal antibody specific to overexpressed receptors on cancer cells. Physicochemical targeting can be achieved through targeting tumor hallmarks or altering the tumor microenvironment which includes pH, temperature, electric charges, enzymes, light irradiation, and magnetic forces, etc. [35,36,37].

Since there are no previous reports on formulation development of TMB, we explored four different methods for preparation of TPL, which were compared and optimized based on formulation feasibility and physical stability [38,39,40]. Even though ethanol and ether injection methods are widely used to prepare liposomes, TMB is poorly soluble in ethanol and ether. Preparation of TPL using a chloroform-based thin film hydration method and a modified hydration method were compared. Precipitation was observed within 2 h using the thin film hydration method while a modified hydration method showed promising stability and entrapment efficiency. This may be attributed to the inner bilayer distribution of TMB because of the osmotic pressure generated by mannitol. A similar finding has been reported by Patel et al. They observed that docetaxel liposomes prepared using the modified hydration method have a higher physical stability in comparison to the thin film hydration method. They proposed that a modified hydration method promotes the distribution of a lipophilic drug within the inner lipid bilayer, which restricts rapid diffusion of the drug to the aqueous phase [41].

The enhanced permeation and retention (EPR) effect is known as passive targeting and is effective especially for the parenteral administered nanoparticles. Drug tends to accumulate around tumor tissue due to leaky vasculature and an abnormal lymphatic drainage system [10]. Conventional liposomes have poor stability due to formulation aspects and are cleared rapidly by the liver and spleen. Thus, a second-generation liposome with stealth technology is desired to prolong blood circulation time and avoid the uptake by reticuloendothelial system (RES), minimizing the side effects and facilitating the accumulation of nanoliposomes in the tumor matrix [10]. Intra-tumor distribution of liposomes is not guaranteed even if nanoparticles have more exposure around tumor tissue. Thus, active target delivery of liposome by ligand–receptor interactions can serve as an effective tool for the uptake of liposomes. EphA2, a protein tyrosine kinase receptor, was found to be overexpressed in melanoma tumor cells [19]. The YSA peptide ligand was used to target BRAF^V600E^-mutated melanoma with overexpressed EphA2 receptors. PEGylated nanoliposomes of TMB prepared by a modified hydration method using a chelating lipid, DOGS-NTA-Ni, allow for the simple and one-step attachment of a 6His-PEG-YSA peptide on liposomal surfaces [24]. YSA peptide was conjugated with 6-histidine through PEG linker. Chemical conjugation of YSA on liposomal surfaces is more tedious and a less efficient approach, with negligible scope of commercialization [42].

Particle size and zeta potential of nanoparticles play an important role, especially for parenteral administration. Different concentrations of 6His-PEG-YSA peptides were used to optimize particle size and zeta potential. The succinyl group of DOGS-NTA-Ni contributes to negative zeta potential of the liposome. Due to the complexation between the YSA target ligand and DOGS-NTA-Ni, the zeta potential changed from negative to positive in a YSA concentration-dependent manner. It was found that 1:2.5 M DOGS-NTA-Ni:YSA showed no further changes in zeta potential, suggesting that YTPL was saturated with YSA. Thus, a 1:2.5 molar ratio of DOGS-NTA-Ni:YSA was used as an optimized ratio for YTPL.

The application of liposomes in commercial use is restricted by physical stability, even though advantages such as biocompatibility and nontoxicity are very attractive [43]. In this paper, physically stable liposomes were prepared by a modified hydration method. However, physical stability was not sufficient enough to keep this formulation in liquid state for long period of time. Therefore, formulation was freeze-dried to convert it into solid state. We also found that drug loading has a significant impact on precipitation. TPL with 1% TMB loading showed better physical stability compared to TPL with a higher drug load. Nevertheless, we observed a very slow but steady increase in drug precipitation from liposomal formulation over the time period. Thus, it was essential to convert the liposomal formulation into powder for reconstitution. Some of the marketed preparations (e.g., doxorubicin liposomes like Doxil^®^ or Myocet^®^) are available in liquid form [44], wherein water-soluble doxorubicin HCl is actively loaded in the aqueous phase of liposome. However, in our case TMB, being a lipophilic molecule, is present in the lipid phase. For liposomes with poor physicochemical stability, spray drying or freeze drying are used to address the stability problem. Freeze drying, also known as lyophilization, is the most commonly used method to dry liposomal dispersions. This technique is widely used for pharmaceuticals to improve their long-term storage stability [45]. AmBisome^®^ and Visudyne^®^ are available as lyophilized liposomal powders [46]. Trehalose is one of the most widely used cryoprotectant and it usually exerts the best protective effect among the disaccharides. Trehalose-based products such as Avastin^®^, and Lucentis^®^ are commercially available. Trehalose has an ability to reduce hygroscopicity so that hydrogen bonds can form easily, has low chemical reactivity, and has a high glass transition temperature (Tg) [47]. Mannitol was used as a carrier for the liposomes and it is generally not advised for stabilizing liposomes because it may separate from a frozen solution or crystallize within the lyophilized cakes [48,49]. Surprisingly, we found that mannitol in combination with trehalose has a positive effect on particle size of reconstituted liposomes. It was reported that the degree of mannitol crystallization during freeze drying can be controlled by processing conditions and chemical composition [50]. Differential scanning calorimetry (DSC) studies can be used for liposome quality control by thermal analysis to determine purity, polymorphic forms, and the melting point of the sample [51]. TMB in liposomes was maintained in a solubilized state since there was no peak at the temperature of pure crystalline TMB.

The release of liposomes is important to evaluate the quality of the formulation as well as to predict in vivo behaviour of a liposomal drug delivery system. In this study, drug release results did not show burst leaking of TMB from the liposomes. Therefore, we anticipate that TMB will be confined within the liposome and will not show any burst release in the blood. However, it showed promising activity in our in vitro studies, indicating that TMB will be released and act after internalization by cancer cells. We expect liposomes to accumulate at tumor site due to the EPR effect, and for complete release of TMB at the tumor site in vivo due to the active-target effect.

Negligible hemolysis was observed even at the therapeutic concentrations of TMB. The blood-to-plasma ratio determines the concentration of the drug in the general circulation and the concentration of the target drug in plasma, which provides an indication of drug the binding to erythrocytes. The result for blood-to-plasma ratios of TMB is around 1 which indicates that TMB is evenly distributed in plasma and red blood cells.

The very low IC_50_ of the TMB liposome confirmed that TMB was released after internalization of TPL into cancer cells. Moreover, the IC_50_ values of TMB, TPL, and YSL are similar in A375 and SK-MEL-28, while in-vivo behavior may vary due to drug distribution and the microenvironment of the tumor. Coumarin-6 was used as a fluorescent probe in the uptake study. The intensity of this fluorescence dye can be correlated with the extent of liposome uptake. YTPL showed higher cell internalization compared to non-targeted liposomes (TPL) because of receptor-mediated uptake. Surprisingly, YTPL presented less uptake in vemurafenib-resistant cell lines, indicating that the expression of EphA2 receptor could be lower in vemurafenib resistant cell lines. This finding is contradictory to a previous study that found vemurafenib-resistant melanoma cell lines had higher EphA2 receptor expression compared to parent cells [22]. Our Western blot analysis result further confirmed our uptake study results. We found that expression of EphA2 was much lower in vemurafenib-resistant melanoma cells compared to parent cells. Since our results contradicted the existing literature, we further studied the cytotoxicity of vemurafenib in parent and resistant cell lines with an EphA2 inhibitor. We found that drug-resistance is not correlated with the expression of the EphA2 receptor using the EphA2 inhibitor AWL-II-41-27.

## 5. Conclusions

We have successfully developed YSA-anchored trametinib-loaded PEGylated nanoliposomes (YTPL) by a modified hydration method, with better entrapment efficiency and stability compared to the thin film hydration method. Moreover, YTPL showed compatibility with red blood cells as well as the desired cytotoxicity to melanoma cells. Metastatic melanoma-specific delivery of MEK inhibitors can be achieved by targeting overexpressed EphA2 receptors in vemurafenib-sensitive cells using nanosized EphA2-targeted PEGylated liposomes. Nevertheless, YTPL is not a viable option for the treatment of vemurafenib-resistant melanoma since TMB has cross-resistance to vemurafenib-resistant cell lines. Moreover, according to our studies, resistant cell lines do not internalize YTPL as much as parental cell lines.

## Figures and Tables

**Table 1:** Methods for the preparation of trametinib (TMB)-loaded PEGylated liposomes (TPL).

Method	Solubility of TMB	Entrapment Efficiency of TPL (1% *w/w* TMB Loading)
**Thin film hydration**	Soluble	51.6%
**Modified hydration**	Soluble	96.2%

**Table 2:** Particle size and zeta potential of YSA-anchored TMB-loaded nanoliposomes (YTPL) and TMB-loaded nanoliposomes (TPL) after freeze drying.

	Particle Size (nm)		Zeta Potential (mV)	
	Before Freeze Drying	After Freeze Drying	Before Freeze Drying	After Freeze Drying
**YTPL (10% trehalose)**	91.20 ± 12.16	159.10 ± 7.50	−0.92 ± 3.27	−4.44 ± 0.49
**Unfiltered TPL**	109.45 ± 9.40	128.40 ± 1.84	−35.55 ± 9.60	−47.30 ± 1.61

**Table 3:** In vitro hemolysis study of TPL at various TMB concentrations.

	TMB Concentration (μg/mL)			
	100	50	10	2
**% Hemolysis**	5.14	3.70	1.03	0

**Table 4:** In vitro cytotoxicity of TMB, TPL, and YTPL in A375 and SK-MEL-28.

IC_50_ (nM)	A375	SK-MEL-28
**TMB**	0.83 ± 0.36	0.74 ± 0.38
**TPL**	0.68 ± 0.15	0.60 ± 0.13
**YTPL**	0.69 ± 0.40	0.77 ± 0.15

## Supplementary Materials

The following are available online at https://www.mdpi.com/1999-4923/11/10/504/s1, S1: Method to estimate YSA peptide, Figure S1: HPLC standard curve of TMB, Figure S2: Cytotoxicity assay in melanoma cell lines.
