# Supplementary material for: EphA2-Receptor Targeted PEGylated Nanoliposomes for the Treatment of BRAFV600E Mutated Parent- and Vemurafenib-Resistant Melanoma
Source: Pharmaceutics. 2019 Oct 1;11(10):504. doi: 10.3390/pharmaceutics11100504 (PMC6836218; doi:10.3390/pharmaceutics11100504)
Supplement: Supplementary file 1 [file pharmaceutics-11-00504-s001.pdf]

pharmaceutics

Supplementary Materials: EphA2-Receptor Targeted PEGylated Nanoliposomes for the Treatment of BRAF^{V600E} Mutated Parent- and Vemurafenib-Resistant Melanoma

Yige Fu, Drishti Rathod, Ehab M. Abo-Ali, Vikas V. Dukhande and Ketan Patel

1. Supplementary File 1

Method: YSA binding was evaluated by BCA protein estimation kit (Thermoscientific, Waltham, MA, USA). Briefly, 400 μL of YSA coated liposomes was filled into Amicon Ultra centrifugal filters (30 kDa) (Millipore, Ireland) and free YSA was separated by centrifuging the samples at 10,000 rpm for 10 min. Concentration of free YSA (before and after freeze drying) was analyzed using a BCA protein estimation kit.

%YSA binding = (amount of YSA attached to liposomes/total YSA added) × 100

Result: YSA binding percentage showed no significant different before and after freeze drying, which shows more than 95% YSA binding on the liposome.

2. Supplementary File 2

Method: The stock solution of Trametinib TMB (100 ppm) was diluted to 20 μg/mL, 10 μg/mL, 5 μg/mL, 2 μg/mL and 1 μg/mL with acetonitrile. The working solution with different concentration TMB were injected in to HPLC with the method described in the manuscript.

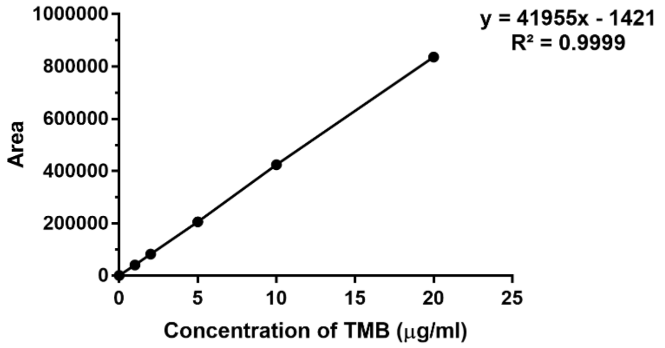

Figure S1. HPLC standard curve of TMB.

3. Supplementary file 3 Cytotoxicity Curves.

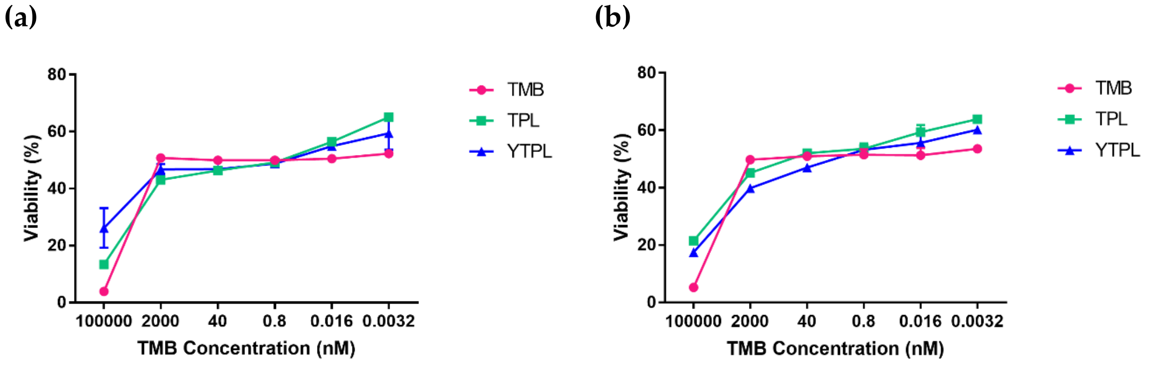

Figure S2. Cytotoxicity assay in melanoma cell lines. % Cell viability of (**a**) A375 cells and (**b**) SK-MEL-28 cells after treatment of TMB, (TMB loaded PEGylated Liposomes) TPL and YTPL (YSA anchored TPL)
